# Relationship between intestinal flora structure and metabolite analysis and immunotherapy efficacy in Chinese NSCLC patients

**DOI:** 10.1111/1759-7714.13442

**Published:** 2020-04-23

**Authors:** Peng Song, Dongliang Yang, Hanping Wang, Xiaoxia Cui, Xiaoyan Si, Xiaotong Zhang, Li Zhang

**Affiliations:** ^1^ Department of Respiratory Medicine, Peking Union Medical College Hospital Chinese Academy of Medical Science & Peking Union Medical College Beijing China; ^2^ Department of General Education Courses Cangzhou Medical College Cangzhou China

**Keywords:** Β‐diversity, intestinal flora, non‐small cell lung cancer, programmed cell death 1 (PD‐1), programmed cell death 1 ligand(PD‐L1)

## Abstract

**Background:**

Many immune checkpoint inhibitors (ICIs) have been approved in China to treat non‐small cell lung cancer (NSCLC). However, in the long term, less than 20% of patients benefit from ICIs. To maximize the benefit for NSCLC patients, it is necessary to guide the choice of immunotherapy through biomarkers. Recent studies have shown that gut microbiota can affect tumor response to immunotherapy and might be a potential predictive biomarker. This study analyzed the relationship between intestinal flora structure and metabolomic characteristics in NSCLC and the efficacy of ICIs.

**Methods:**

Prospective analysis of samples from 63 patients with advanced NSCLC who attended the Department of Respiratory Medicine of the Peking Union Medical College Hospital from March 2018 to June 2019, and were prescribed programmed cell death 1 (PD‐1) inhibitors, was carried out. The follow‐up deadline was 31 December 2019. Stool samples were collected from all patients before the start of immunotherapy. DNA was extracted from all samples and libraries were constructed. This was followed by sequencing using the Illumina sequencing platform, and results were studied using a biological information data analysis process. We divided the data into two groups based on progression‐free survival (PFS) ≥ six months and PFS < six months.

**Results:**

The median PFS was 7.0 months, not reaching the median overall survival (OS). We obtained 373.5 G of original sequencing data. The phyla Bacteroidetes, Firmicutes, Proteobacteria, and Actinobacteria accounted for most of the bacterial communities in the stool samples studied. Compared with the PFS < six‐month group, the patients in the PFS ≥ six‐month group had significantly higher β‐diversity in the intestinal microbiome at the baseline level. There were also differences in composition between the two groups. Samples in the PFS ≥ six‐month group were rich in *Parabacteroides* and *Methanobrevibacter*, while those in the PFS < six‐month group were rich in *Veillonella*, Selenomonadales, and Negativicutes. The KO, COG, and CAZy databases were used to study functional group protein families, yielding 390 (KO), 264 (COG), and 859 (CAZy) functional group abundances, with significant differences between the two groups. Bacterial metabolites analysis suggested significant differences in the metabolic potential of methanol and methane between the two groups.

**Conclusions:**

We found a close correlation between intestinal microbiome β‐diversity and anti‐PD‐1 immunotherapy response in Chinese patients with advanced NSCLC. The intestinal flora composition, functional group protein family, and KEGG metabolism also differed between the two groups. Differences in pathways and flora metabolites were also noted.

## Introduction

Non‐small cell lung cancer (NSCLC) accounts for 85% of diagnosed lung cancers. About 50% of NSCLC patients are diagnosed when they are already at stage IV, and their five‐year survival rate is less than 10%.^1^ The emergence of immune checkpoint inhibitors (ICIs) targeting programmed cell death 1 (PD‐1), or its ligand (PD‐L1) has significantly changed the treatment and management of locally advanced and advanced NSCLC. Multiple randomized controlled trials (RCTs) have shown that ICIs are superior to docetaxel^2^, ^3^, ^4^ as a second‐line treatment for advanced NSCLC patients. ICIs have been approved by the US Food and Drug Administration (FDA) to treat patients with 15 different cancer types.^5^ However, most tumors appear to lack T cell infiltration and active expression of immune genes. Only 20% of patients with advanced NSCLC benefit from treatment, while up to 50% of patients experience treatment‐related adverse events. Considering the high cost of the drug, the limited population that benefits from it, and the potential serious side effects, it is important to explore biomarkers for selecting patients who might benefit from ICI treatment in advanced NSCLC. Recent studies have shown that the gut microbiota could influence tumor response to immunotherapy and might be a potential predictive biomarker. However, published studies have focused on populations in the Western world. Noting that the structure of the intestinal microbiome differs significantly between different races,^6^ and that geographic location, diet, lifestyle, and genetic factors can all contribute to this difference,^7^, ^8^ we aimed to study the association between the microbiome and immunotherapy in Chinese NSCLC patients.

## Methods

### Patients

We prospectively analyzed samples from 63 patients with advanced NSCLC who visited the Peking Union Medical College Hospital from March 2018 to June 2019 and were prescribed PD‐1 or PD‐L1 inhibitors. According to the response evaluation criteria in solid tumor (RECIST) 1.1 standard, progression‐free survival (PFS) is defined as the time from the start of immunotherapy to the progression of the disease or death of the patient. Overall survival (OS) is defined as the time interval between the initiation of immunotherapy and the time of death or the final follow‐up. Efficacy is determined every six to eight weeks after the start of the immunotherapy. In special cases, the time interval can be adjusted to suit the patients' needs. The enrollment deadline for patients in our study was 30 June 2019, and the follow‐up deadline was 31 December 2019. The Ethics Committee of the Peking Union Medical College Hospital approved the study, which is in line with the ethical principles of the Helsinki Declaration. All patients provided their signed informed consent.

### Sample collection

Before the start of immunotherapy, the patient used a special sampler to collect a stool sample (the first defecation sample in the morning). The size of the sample was in the range of broad bean grains. Samples were stored in a protective solution at room temperature and sequenced as soon as possible. We strived to use the inner part of the stool samples for sequencing to avoid environmental factors contamination.

### DNA extraction and sequencing

The DNeasyPowerSoilKit was used to extract fecal microbial genomic DNA. Extracted samples were stored at −20°C pending further evaluation. The NanoDrop ND‐100 spectrophotometer and agarose gel electrophoresis were used to determine the quantity and quality of the extracted DNA, respectively. We used the Illumina TruSeq Nano DNA LT Library Preparation Kit to process the extracted DNA to construct a 400 bp metagenomic shotgun sequencing library. We then used the Illumina HiSeq sequencing platform to sequence the library.

### Statistical analysis

Descriptive statistical analysis was used to summarize the clinicopathological features. Reports on continuous variables used the median and quartile ranges, while we describe the frequency of distribution for categorical variables. We applied the metagenomic sequencing for intestinal flora analysis. The analysis included quality control, assembly, gene prediction, construction of nonredundant gene sets, gene function annotation, quantitative abundance at the gene, species, and functional levels, and flora composition, diversity, multidimensional difference, functional, and metabolite analyses.

## Results

### Patient characteristics

Table 1 summarizes the demographic, clinical, and pathological characteristics of the 63 NSCLC patients. The median age of the population was 61 years (39–81 years). A total of 36 patients (57.14%) were over the age of 60 years. There were 53 males (84.13%) and 10 females (15.87%). A total of 58 patients (92.07%) had an Eastern Cooperation Oncology Group (ECOG) performance score ≤ 1, suggesting that the analysis of patients' physical fitness was good. The proportion of adenocarcinoma (42.86%) was lower than that of squamous cell carcinoma (57.14%). Most patients were at stage IV of the disease (84.12%) at the beginning of the immunotherapy. The medications used in the patients included pembrolizumab in 42 cases, nivolumab in four cases, and sintilimab in 17 cases.

**Table 1:** The demographic, clinical, and pathological characteristics of the patients

	Cohort	
Patient characteristics	No. of patients	%
**Age**		
≤60	27	42.86
>60	36	57.14
**Gender**		
Female	53	84.13
Male	10	15.87
**ECOG scores**		
0	20	31.75
1	38	60.32
2	5	7.93
**Pathology**		
Squamous cell carcinoma	36	57.14
Adenocarcinoma	27	42.86
**TNM stage**		
IIIB	4	6.35
IIIC	6	9.52
IVA	39	61.90
IVB	14	22.22
**Immunotherapy lines**		
1	32	50.79
2	22	34.92
≥3	9	14.29

### Efficacy analysis

The follow‐up deadline was 31 December 2019. A total of 63 patients were included in the statistical analysis. The best effect was a partial response (PR) in 27 cases (42.8%), stable disease (SD) in 14 cases (22.2%), and progressive disease (PD) in 22 cases (34.9%). The objective response rate (ORR) was 42.8%, and the disease control rate (DCR) was 65.1%. There were 50 cases of disease progression, accounting for 79.4%. Those who did not show disease progression (*n* = 13, 20.6%) were regarded as censored data. A total of 14 patients died during the study period, accounting for 22.2%. The median PFS was 7.0 months (95% CI: 5.0–11.0 months), and the median OS was not reached. There were 35 patients with PFS ≥ six months and 28 patients with PFS < six months.

### Species accumulation curve

DNA, extracted from 63 stool samples, was sequenced. The number of reads ranged between 27 060 458–66 836 852, with an average of 43 119 830 reads. All samples were included in the biological information data analysis process.

**Figure 1:**
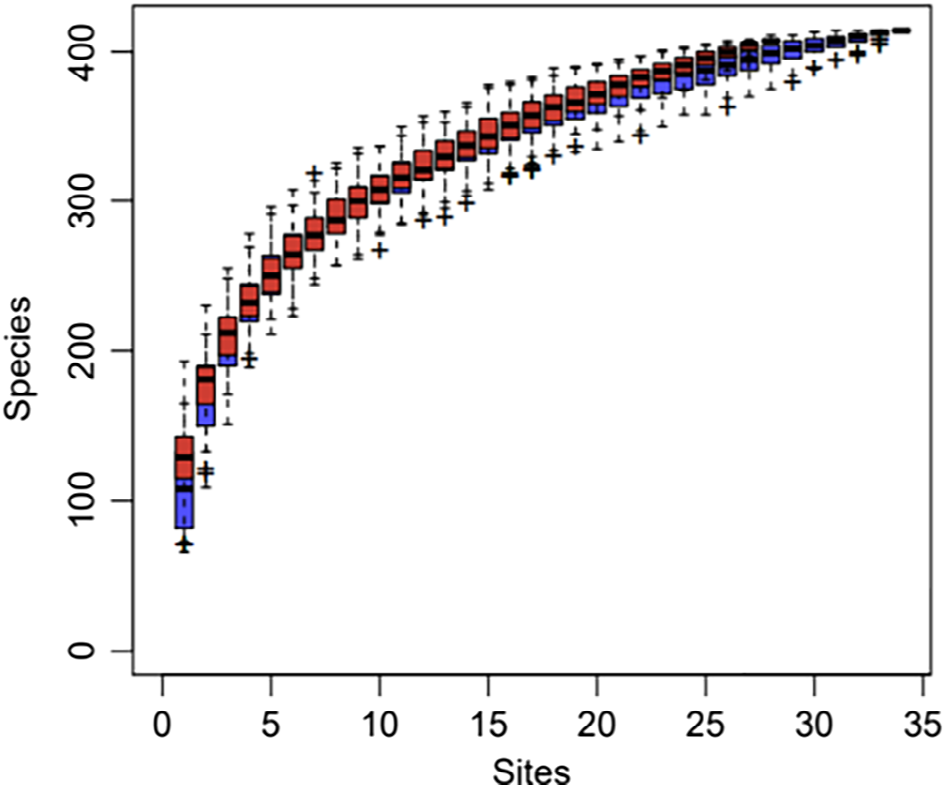
Comparison of species accumulation curves between groups. (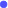) PFS_gt_6mo, (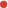) PFS_it_6mo

Species accumulation curves were used to describe the increase in species with the increase in sample size. This approach can determine the sufficiency of the sample size and estimate species richness. The species accumulation curves in the vegan function specaccumin R was used for this purpose. A comparison between the curves obtained for the two groups, based on the relative abundance of the flora, is shown in Fig 1. The upward trend at the end of the curve became flattened, indicating that the sample size was sufficiently large and that new species could not be discovered by increasing the sample size.

### Diversity analysis

**Figure 2:**
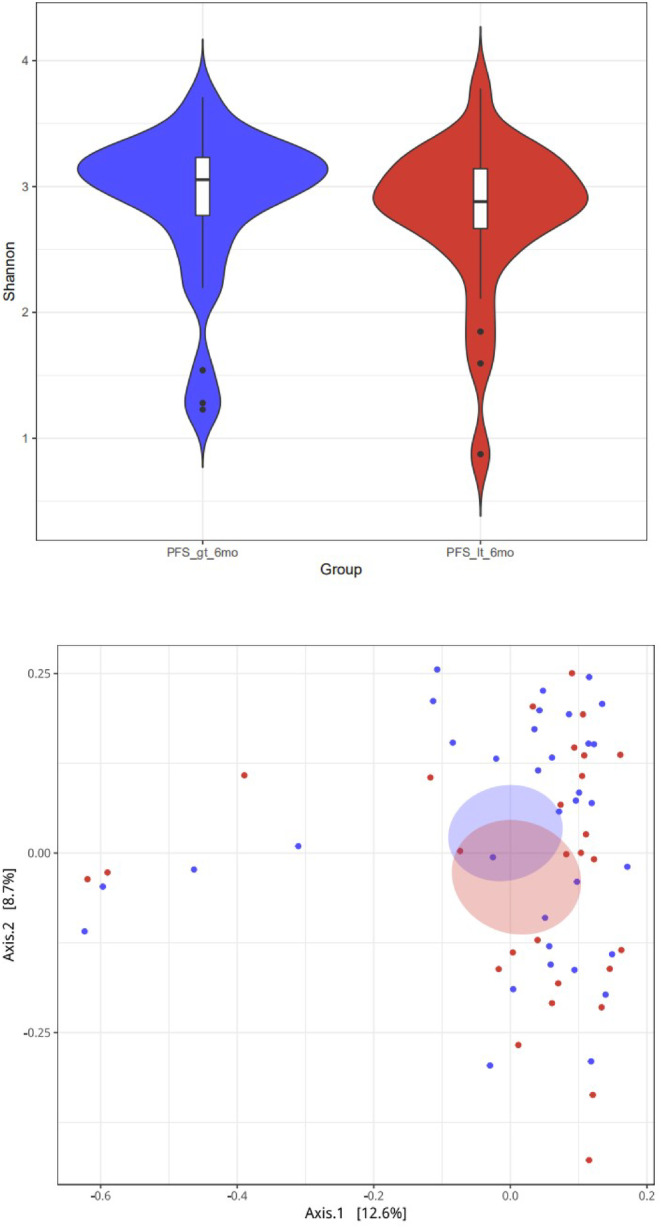
(**a**) Comparison of α diversity of different groups at the flora level. Group (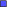) PFS_gt_6mo, (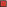) PFS_it_6mo (**b**) PCoA results based on the sample distance calculated by the flora (Note: PFS_gt_6 mo = PFS ≥ six‐months, PFS_it_6mo = PFS < six‐months). Group (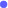) PFS_gt_6mo, (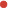) PFS_it_6mo

The α‐diversity (richness, uniformity, Shannon index of diversity, etc) is an indicator describing species diversity with in a sample. One can calculate these indicators, using the diversity function in the R package. In this study, we used the Shannon index to represent the α‐diversity of the samples. The α‐diversity of the intestinal microbiome was higher in the PFS ≥ six‐month group than in the PFS < six‐month group (Fig 2a), but the difference was not significant (*P* = 0.12). The principal coordinate analysis (PCoA) method, based on the Bray‐Curtis distance, was used to represent the β‐diversity of the samples. The similarity and difference between samples were displayed on two‐dimensional coordinates. There were significant differences in β‐diversity between the two groups. As shown in Fig 2b, the baseline samples are clustered by the response status of the PFS. Components 1 and 2 accounted for 12.6% and 8.7% of the difference in PCoA, respectively.

### Analysis of intestinal flora

**Figure 3:**
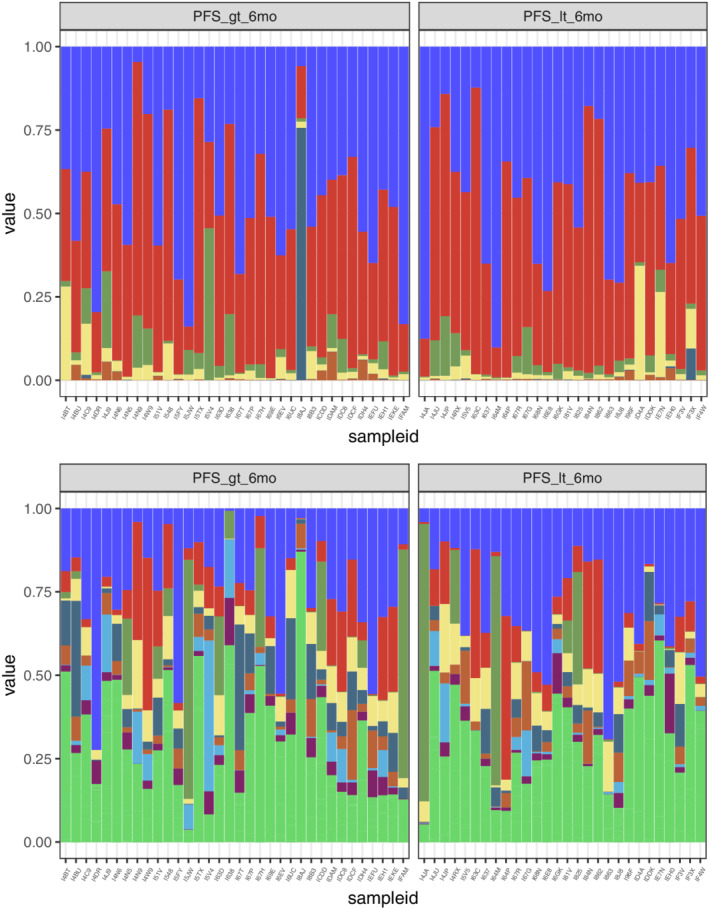
Relative abundance composition of different groups at the Phylum and Genus levels (Note: PFS_gt_6mo = PFS ≥ six‐months, PFS_it_6mo = PFS < six‐months). (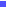) phylum p_Bacteroidetes, (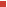) p_Firmicutes, (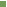) p_Actinobacteria, (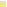) p_Proteobacteria, (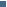) p_Viruses_noname, (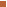) other; genus (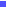) g_Bacteroides, (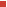) g_Eubacterium, (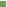) g_Prevotella, (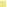) g_Faecalibacterium, (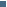) g_Alistipes, (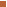) g_Ruminococcus, (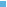) g_Bifidobacterium, (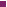) g_parabacteroides, (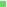) other

We calculated the relative abundance of the intestinal bacterial composition at the phylum, order, family, genus, and species levels between the PFS ≥ six‐month and the PFS < six‐month groups. At the gene level, high‐quality reads were compared to the constructed nonredundant reference gene set by the Burrows‐Wheeler aligner (BWA) software. Reads of less than 30 bp long or less than 95% in consistency were removed to obtain clean reads count for all genes that were then standardized. The relative abundance of the genes was obtained by processing. At the species level and other advanced taxonomy, MetaPhlAn2 was used to obtain the relative abundance of the intestinal flora from the species to the phylum levels. MetaPhlAnv2.0 uses over one million marker genes from an average of 7500 species (on average, 184 markers per species) to make predictions and obtain relative abundance at the different classification levels. Bacteroidetes, Firmicutes, Proteobacteria, and Actinobacteria accounted for most of the bacterial communities in the stool samples studied (Fig 3).

### Analysis of differences between groups

**Figure 4:**
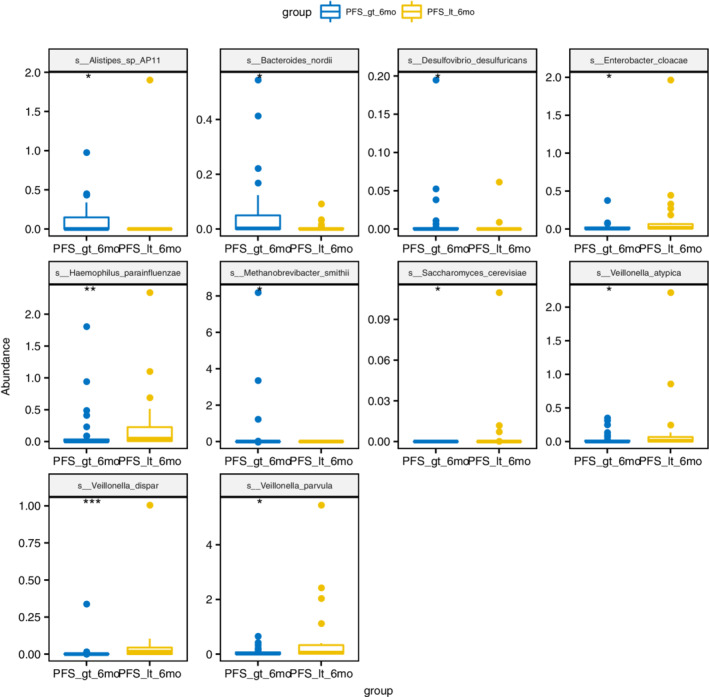
Box comparison chart of significantly different bacteria (TOP10, sorted by *P*‐value) (Note: PFS_gt_6mo = PFS ≥ six‐months, PFS_it_6mo = PFS < six‐months). group (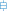) PFS_gt_6mo, (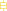) PFS_it_6mo

The Wilcoxon rank‐sum permutation test was used to examine the differences in the relative abundance of bacteria between the groups at the phylum, class, order, family, genus, and species levels. There were no significant differences at the phylum level between the groups. At the class level, Methanobacteria was enriched in the PFS ≥ six‐month group (*P* = 0.037), and Negativicutes was enriched in the PFS < six‐month group (*P* = 0.049). At the order level, Methanobacteriales (*P* = 0.037) were enriched in the PFS ≥ six‐month group. Pasteurellales (*P* = 0.007), Pseudomonadales (*P* = 0.022) and Selenomonadales (*P* = 0.049) were enriched in the PFS < six‐month group. At the family level, Methanobacteriaceae (*P* = 0.037) was enriched in the PFS ≥ six‐month group. Veillonellaceae (*P* = 0.006) Pasteurellaceae (*P* = 0.007) and Pseudomonadaceae (*P* = 0.022) were enriched in the PFS < six‐month group. At the Genus levels, Desulfovibrio (*P* = 0.006), Holdemania (*P* = 0.037), Methanobrevibacter (*P* = 0.037) and Parabacteroides (*P* = 0.040) were enriched in groups with PFS ≥ six‐month, Veillonella (*P* = 0.005), Haemophilus (*P* = 0.008), Pseudomonas (*P* = 0.022), Butyrivibrio (*P* = 0.022), Saccharomyces (*P* = 0.025), Enterobacter (*P* = 0.029) and Leuconostoc (*P* = 0.031) were enriched in the group with PFS < six‐month. A total of 15 species of intestinal bacterial abundance were statistically different at the Species level. Alistipes_sp_AP11 (*P* = 0.022), Desulfovibrio_desulfuricans (*P* = 0.024), Bacteroides_nordii (*P* = 0.030), Methanobrevibacter_smithii (*P* = 0.037), Blautia_hydrogenotrophica (*P* = 0.037), Lactobacillus_mucosae (*P* = 0.041), Parabacteroides_merdae (*P* = 0.047) were enriched in the PFS ≥ six‐month group,Veillonella_dispar (*P* = 0.0002), Haemophilus_parainfluenzae (*P* = 0.006), Enterobacter_cloacae (*P* = 0.011), Saccharomyces_cerevisiae (*P* = 0.025), Veillonella_atypica (*P* = 0.026), Veillonella_parvula (*P* = 0.031), Gemella_haemolysans (*P* = 0.040), Paraprevotella_clara (*P* = 0.044) were enriched in the PFS < six‐month Group. Fig 4 shows a box comparison chart of the top 10 significantly different bacteria at the Species level.

### LEfSe analysis

**Figure 5:**
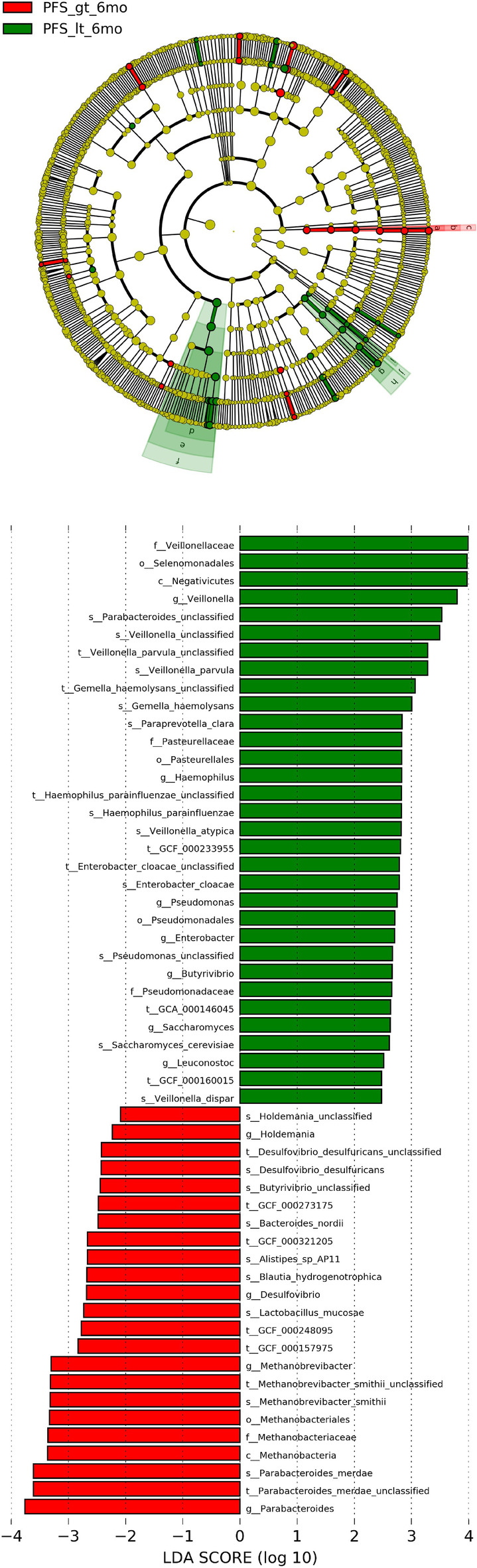
Linear discriminant analysis (LDA) scores for different groups. The LEfSe classification results and classification chart show the differences in fecal taxa. The size of the dots is proportional to the abundance of the taxon. LDA scores of the different taxa in the stool microbiome of PFS ≥ six months (red) and PFS < six months (blue); the length indicates the size of the effect related to the taxonomy, LDA score > 3 is significant. (Note: PFS_gt_6mo = PFS ≥ six‐months, PFS_it_6mo = PFS < six‐months). (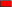) PFS_gt_6mo, (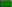) PFS_it_6mo, (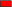) Methanobacteriaceae, (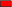) Methanobacteriales, (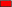) Methanobrevibacteria, (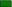) Veillonellaceae, (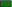) Selenomonadales, (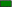) Negativicutes, (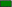) Pasteurellaceae, (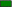) Pasteurellales, (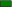) Pseudomonadaceae, (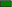) Pseudomonadales

To further explore these findings, we performed a high‐dimensional classification comparison, using linear discriminant analysis of effect size (LEfSe), to find biomarkers with significant differences in abundance between the groups. This analysis proves again that the bacteria in the fecal microbiome of the PFS≥six‐month group and the PFS < six‐month group differed significantly in their response to PD‐1 treatment. We observed the overexpression of several bacterial genera (Fig 2b). Relative to patients with PFS < six months, the most significantly related flora in patients with PFS ≥ six months were *Parabacteroides* (LDA score = 3.8) and *Methanobacteriaceae* (LDA score = 3.4). The PFS < six‐month group was rich in *Veillonella*, *Selenomonadales*, and *Negativicutes* (Fig 5).

### Relative functions and differences of microorganisms

Based on gene addition read count abundance of each functional level, the relative abundance of each functional group protein families between groups (based on KO, COG, and CAZy databases) was analyzed using DESeq2.

The results suggest that there were 390 (KO), 264 (COG), and 859 (CAZy) functional group abundances with significant differences between the groups. Table 2 shows the top 20 relative abundance KOs with significant differences between the groups. Of these, 12, including K20947, were significantly higher in the PFS ≥ six‐month group and eight, including K09628, were significantly higher in the PFS < six‐month group. Table 3 shows the top 20 relative abundances of CAZy with significant differences between the groups. Of these, 16, including GH43, were significantly higher in the PFS ≥ six‐month group and four, including PL6, were significantly higher in the PFS < six‐month group. Table 4 shows the top 20 relative abundance COGs with significant differences between the groups. Of these, 16, including COG1469, were significantly higher in the PFS ≥ six‐month group and four, including COG285, were significantly higher in the PFS < six‐month group.

**Table 2:** KEGG Orthology difference results (top 20)

ID	PFS ≥ 6 months	PFS < 6 months	log2(fold change)	Stat	*P*‐value	Padj
K20947	33.559	8.107	−4.541	−7.448	0.000	0.000
K09628	3.676	71.821	4.162	7.338	0.000	0.000
K17589	42.912	25.393	−4.652	−7.253	0.000	0.000
K14340	28.647	1.786	−3.880	−6.621	0.000	0.000
K14053	4.382	44.393	3.351	6.606	0.000	0.000
K08794	170.971	108.071	−3.488	−6.471	0.000	0.000
K17916	89.265	8.857	−3.929	−6.383	0.000	0.000
K11005	113.088	50.214	−3.458	−6.296	0.000	0.000
K08604	21.176	52.429	−3.294	−5.866	0.000	0.000
K11366	4.235	58.857	3.682	5.804	0.000	0.000
K00064	27.647	5.857	−3.219	−5.798	0.000	0.000
K09489	3.235	47.393	3.645	5.784	0.000	0.000
K20034	16.941	3.464	−3.098	−5.736	0.000	0.000
K19626	36.500	2.679	−3.663	−5.724	0.000	0.000
K17346	1.059	6.357	2.722	5.677	0.000	0.000
K16766	17.294	1.964	−3.216	−5.624	0.000	0.000
K03252	5.765	70.000	3.428	5.558	0.000	0.000
K09882	289.324	56.286	−2.335	−5.554	0.000	0.000
K03219	58.265	14.036	−2.977	−5.531	0.000	0.000
K15839	21.441	21.964	−3.193	−5.508	0.000	0.000

**Table 3:** CAZy difference results (top 20)

ID	PFS ≥ 6 months	PFS < 6 months	W	*P*‐value
GH43	134.48	8.04	731.00	0.00
GT83	18.30	7.81	692.00	0.00
CBM67	17.82	4.53	676.00	0.00
PL6	1.74	380.54	304.50	0.00
GT2	335.57	76.37	721.00	0.00
GT83	241.29	722.74	231.50	0.00
CE2	13.96	0.26	660.00	0.00
GH109	2783.22	1829.32	708.00	0.00
CE4	243.93	15.40	680.50	0.00
GH39	102.85	5.38	662.00	0.00
GT4	218.58	27.15	681.50	0.00
GH43	0.12	21.44	332.50	0.00
CE4	120.36	4.39	639.50	0.00
GH43	270.99	45.63	688.00	0.00
CBM32	41.23	205.84	263.50	0.00
GT8	24.43	0.89	644.00	0.00
GH2	35.33	6.61	647.00	0.00
GH130	340.38	25.84	672.00	0.00
GH92	17.65	7.70	634.50	0.00
GH31	30.46	17.04	668.00	0.00

**Table 4:** COG difference results (top 20)

ID	PFS ≥ 6 months	PFS < 6 months	W	*P*‐value
COG2895	12 210.93	16 697.74	219	0.00
COG1469	1182.13	621.98	718	0.00
COG3256	120.60	493.48	233	0.00
COG2108	562.79	152.66	716	0.00
COG4290	3398.93	2589.59	707	0.00
COG2519	1974.17	1056.65	702	0.00
COG3269	795.27	233.00	706	0.00
COG1545	165.72	26.02	689	0.00
COG5607	284.18	101.66	696	0.00
COG1355	117.00	9.57	656	0.00
COG2212	873.03	452.81	691	0.00
COG0328	13 014.18	9139.23	685	0.00
COG1635	2315.64	1299.39	684	0.00
COG1751	120.97	72.51	676	0.00
COG3717	16 098.02	21 052.69	270	0.00
COG1833	186.25	624.31	268	0.00
COG1320	929.23	475.73	684	0.00
COG2111	1453.55	759.97	684	0.00
COG1537	58.90	1.03	632	0.00
COG1006	950.30	540.52	683	0.00

### Enrichment analysis of KEGG metabolic pathways

According to the analysis of intestinal microbial metagenomics in the two groups, the relative abundance distribution table of KO functional groups was obtained in the KEGG function database. The KO pathway (TOP10) with a significant difference in relative abundance between the two groups was analyzed. The results of pathway enrichment analysis showed (Table 5) that the two groups differed in 21 pathways. Compared with the PFS ≥ six‐month group, in the PFS < six‐month group ko00680 methane metabolism ko00362 Benzoate degradation, ko03070 Bacterial secretion system, ko00622 Xylene degradation, Ko03008 Ribosome biogenesis in eukaryotes and other three metabolic pathways were downregulated, ko02040 Flagellar assembly, ko02030 Bacterial chemotaxis, ko00052 Galactose metabolism, Ko02020 Two‐component system, ko04020 Calcium signaling pathway and other eight metabolic pathways were upregulated.

**Table 5:** KEGG metabolic pathway enrichment analysis

KEGG pathway	*P*‐value geomean	Statistical mean	*P*‐value	Set. size	Regulation
ko00680 methane metabolism	0.00	−5.40	0.00	120.00	Down
ko02040 flagellar assembly	0.00	4.74	0.00	37.00	Up
ko02030 bacterial chemotaxis	0.00	4.59	0.00	26.00	Up
ko00362 benzoate degradation	0.00	−3.36	0.00	50.00	Down
ko00052 galactose metabolism	0.00	3.09	0.00	54.00	Up
ko02020 two‐component system	0.00	2.92	0.00	383.00	Up
ko03070 bacterial secretion system	0.01	−2.37	0.01	69.00	Down
ko00622 xylene degradation	0.01	−2.49	0.01	13.00	Down
ko04020 calcium signaling pathway	0.01	2.42	0.01	14.00	Up
ko03008 ribosome biogenesis in eukaryotes	0.01	−2.31	0.01	34.00	Down
ko02024 quorum sensing	0.01	2.24	0.01	167.00	Up
ko02026 biofilm formation ‐ *Escherichia coli*	0.02	2.14	0.02	50.00	Up
ko00627 aminobenzoate degradation	0.02	−2.11	0.02	30.00	Down
ko03010 ribosome	0.03	−1.91	0.03	92.00	Down
ko00540 lipopolysaccharide biosynthesis	0.03	1.93	0.03	40.00	Up
ko03020 RNA polymerase	0.03	−1.91	0.03	23.00	Down
ko04727 GABAergic synapse	0.04	1.84	0.04	12.00	Up
ko00520 amino sugar and nucleotide sugar metabolism	0.04	1.76	0.04	120.00	Up
ko02060 phosphotransferase system (PTS)	0.04	1.76	0.04	71.00	Up
ko04024 cAMP signaling pathway	0.04	1.80	0.04	17.00	Up
ko02010 ABC transporters	0.04	1.71	0.04	369.00	Up

### Metabolite analysis

The gut flora can produce and consume many metabolites. We estimated the potential of the intestinal flora to generate or consume certain metabolites by accumulating the abundance of the flora that produces such metabolites and subtracting the abundance of the flora that consumes them. This assisted with the estimation of the difference in metabolic potential between groups. The results (Table 6) suggest that the potential for methane metabolism in the PFS ≥ six‐month group was higher than that in the PFS < six‐month group (*P* = 0.04), while the potential for methanol metabolism was lower than that in the PFS < six‐month group (*P* = 0.03). There was no difference in the remaining metabolites.

**Table 6:** Differences in metabolite potential between groups

Metabolites	PFS ≥ 6 months	PFS < 6 months	W	*P*‐value
Methanol	1.06	3.22	321	0.03
Methane	0.38	0.00	546	0.04
Polypeptide	2.71	4.53	342	0.06
Cadaverine	1.34	2.51	357	0.09
Menaquinone	0.93	4.11	366	0.12
Formate	34.92	41.75	372	0.14
Putrescine	2.63	3.60	372	0.14
L‐Threonine	1.28	2.37	373	0.15
Trimethylamine	8.01	9.90	376	0.16

## Discussion

The human microbiome is a complex and diverse ecosystem,comprising bacteria, archaea, virions (phages and eukaryotic viruses), fungi, and fauna (single‐cell protozoa and worms).^9^ This microbiome can be analyzed genetically and is affected by the environment. The integrity of the intestinal mucosal barrier is important for the development and cohomeostasis of the immune system.^10^ With continuous progress in high‐throughput sequencing technology, we can now better understand the intestinal flora. The human intestine contains 10^13^ microorganisms, which are 10 times more numerous than the host cells. Changes in the structure and function of the intestinal flora are closely related to the physiological and pathological processes of the host. The intestinal flora is called the new “organ” of the human body and is used as a new “target” for drug development. Many studies have shown that intestinal flora disorders are closely related to the occurrence and development of obesity, diabetes, and hyperlipidemia.^11^, ^12^ Recent studies have also shown that the gut microbiota can influence tumors' response to immunotherapy and might thus be a potential predictive biomarker. However, research has thus far focused only on populations in the Western world.

In this study, Chinese NSCLC patients with PFS ≥ six months and PFS < six months treated with immunotherapy, were compared for differences in the composition and function of their fecal specimens, using the metagenomic sequencing technology. First, we performed a diversity analysis of the two groups of patients, which showed that the α‐diversity of the intestinal microbiome was higher in the PFS ≥ six‐month group. This difference, however, was not statistically significant. There were, however, significant differences in β‐diversity between the groups. Components 1 and 2 accounted for 12.6% and 8.7% of the difference in PCoA, respectively. Jin *et al*.^13^ grouped 25 patients who used nivolumab according to responders (R) and nonresponders (NR) and found that the α‐diversity of baseline gut microbiome was significantly higher in the R group (*n* = 13) than in the NR group (*n* = 12). Significant differences in β‐diversity were also observed between the R and NR groups. Routy *et al*.,^14^ Gopalakrishnan *et al*.,^15^ and Matson *et al*.^16^ also found the same phenomenon in lung cancer, renal cell carcinoma, and melanoma patients. The lack of statistically significant differences between the groups in the α‐diversity of the bacterial flora in this study might be related to the type of immunotherapy drugs used. This aspect needs to be explored further.

This study found that the most significantly related flora in patients with PFS ≥ six months was Parabacteroides and Methanobacteriaceae. In patients with PFS < six months, the flora was enriched in Veillonella, Selenomonadales, and Negativicutes. Parabacteroides is one of the core human flora. Studies have shown that its content is negatively related to obesity, nonalcoholic fatty liver, and diabetes. It can significantly reduce obesity, induced in mice by high‐fat diets. It can also decrease insulin resistance, lipid metabolism disorders, and nonalcoholic fatty liver symptoms. Parabacteroides have a wide range of bile acid conversion functions. They can also activate intestinal gluconeogenesis, and thereby regulate appetite, promote liver glycogen synthesis, and improve host glucose metabolism disorders. *Paracobacterium* sp. produces succinic acid and secondary bile acid by activating different signaling pathways. Through these, it exerts overall multitarget regulation and is a potential new type of antimetabolic syndrome probiotic.^17^ Some researchers have confirmed that anaerobic glycolysis inside tumor cells depletes extracellular glucose, which in turn limits T cell glucose utilization.^18^ By improving the host's glucose metabolism disorders, Parabacteroides might also improve the efficacy of immunotherapy. Methanobacter is an archaeon that uses methane and carbon dioxide as specific metabolites under anaerobic conditions. With a unique 16S rRNA oligonucleotide sequence profile, their cell wall does not have muramic acid and D‐type amino acids common to other bacterial cell walls. The possible mechanisms activated by Methanobacter during immunotherapy of lung cancer is currently unknown, and further research is needed to elucidate this aspect.

Four recent studies have confirmed the effect of intestinal flora on ICIs in cancer patients. In the study by Routy *et al*.^14^ metagenome sequencing analysis in patients with advanced lung, renal, and urothelial cancer, who received anti‐PD1 treatment, showed that, compared with nonresponders, stool cultures from responding lung cancer patients showed significantly higher mucus production. These studies also emphasized that the frequency of hemolytic *Staphylococcus* and *Corynebacterium* were higher in patients with NR, and that *Enterococcus pneumonia* was more represented in patients with R. Gene sequencing analysis of fecal samples from 43 patients based on 16S rRNA done by Gopalakrishnan *et al*.^15^ found that flora in NR patients was enriched in *Clostridium*, while samples from patients in R group were enriched in Ruminococcaceae and *Faecalibacterium* (RECIST1.1 or a six‐month stabilization period). Shotgun metagenomics on 25 samples from the same cohort confirmed the enrichment of *Feacalibacterium* species in patients with type R. Matson *et al*.^16^ reported analysis results of 38 fecal pretreatment samples, showing that *Bifidobacterium longum*, *Collinsella aerofaciens*, and *Enterococcus faecium* were associated with a better prognosis. Jin *et al*.^13^ performed shotgun metagenomic sequencing on 25 Chinese patients and found that unclassified *Ruminococcus* was enriched in the NR group, while *Alistipesputredinis*, *Prevotella*, *Bifidobacterium longum*, *Lachnobacterium*, Lachnospiraceae, and *Shigella* were enriched in R group. Taken together, these studies confirm that the gut flora can modulate the response to PD‐1 / PD‐L1 inhibitors. However, it is undeniable that the above five studies have identified different bacterial signals (*Parabacteroides*, *Akkermansia*, *Faecalibacterium*, *Bifidobacterium*, and *Alistipesputredinis*) related to PD‐1 inhibitor response. After all, many factors affect the composition of the human microbiome. Examples include diet, early childhood exposure, stress, and long‐term use of antibiotics and other medications.^19^ Therefore, the differences might be partly due to geographical factors of the small sample‐size patient populations and the different criteria for treatment response used in these studies. In addition, the accuracy of shotgun metagenomics for taxonomic identification is limited by the incomplete database of bacterial genome sequences, coupled with the cultivation of clinical bacterial isolates that can identify new species and differentiate strains of the same species. Moreover, the effect of the microbiome on treatment is unlikely to be attributed to a single species, but rather to the gut flora ecology and the combined metabolism that elicit different responses to immunotherapy. The identified species or group of species might constitute biomarkers for these, more complex, ecological changes. Due to the small and heterogeneous populations analyzed, different species might have had important implications for immunotherapy in these five studies.

Functional analysis of intestinal microbial metagenomes in lung cancer immunotherapy in China is currently lacking. This study found that the main functional categories in the PFS ≥ six‐month group were methane metabolism, benzoate degradation, bacterial secretion system, xylene degradation, and true ribosome synthesis in nuclear organisms. The main functional categories in the PFS < six‐month group were bacterial flagellar assembly, bacterial chemotaxis, galactose metabolism, bacterial two‐component regulation system, and calcium signaling pathway. A comparison of metagenomics showed that there were 390 (KO), 264 (COG), and 859 (CAZy) functional groups that significantly differed between the two study groups. The composition structure and function of the intestinal microbiomeare the result of the long‐term co‐evolution of microorganisms and humans. To deepen the understanding of the role of intestinal flora populations in relation to immunotherapy, a deeper analysis of metabolic and related pathways should be conducted.

This study had certain limitations. The theoretical intestinal flora differences were obtained based only on patient populations. They were not confirmed in animal models, nor have they been explored for specific relationships between the immune microenvironment and the intestinal flora. The mechanism of flora immune regulation is also not clear, and a larger sample size will be needed for deeper exploration in the future.

## Disclosure

The authors report that there are no conflicts of interest.
